# Evaluating the effects of mefenoxam on taxonomic and functional dynamics of nontarget fungal communities during carrot cultivation

**DOI:** 10.1038/s41598-024-59587-2

**Published:** 2024-04-29

**Authors:** Setu Bazie Tagele, Emma W. Gachomo

**Affiliations:** Department of Microbiology and Plant Pathology, University of California Riverside, Riverside, CA 92507 USA

**Keywords:** Ecology, Microbial ecology

## Abstract

Ridomil Gold SL (45.3% a.i. mefenoxam) is a widely used chemical fungicide for the control of oomycetes. However, its impact on fungal communities remains unexplored. Therefore, the goal of this study was to examine the effects of mefenoxam on the temporal dynamics of fungal taxonomic and functional diversities during carrot cultivation under four treatment groups: mefenoxam application with and without *Pythium* inoculation, and untreated control groups with and without *Pythium* inoculation. Our in vitro sensitivity assay showed that the maximum recommended concentration of mefenoxam, 0.24 ppm, did not suppress the mycelial growth of *P. irregulare*. At 100 ppm, mycelial growth was only reduced by 11.4%, indicating that the isolate was resistant to mefenoxam. MiSeq sequencing data revealed transient taxonomic variations among treatments 2 weeks post-treatment. *Mortierella* dominated the fungal community in the mefenoxam-*Pythium* combination treatment, as confirmed through PCR using our newly designed *Mortierella*-specific primers. Conversely, mefenoxam-*Pythium* combination had adverse effects on *Penicillium*, *Trichoderma*, and *Fusarium,* and decrease the overall alpha diversity. However, these compositional changes gradually reverted to those observed in the control by the 12th week. The predicted ecological functions of fungal communities in all *Pythium* and mefenoxam treatments shifted, leading to a decrease in symbiotrophs and plant pathogen functional groups. Moreover, the community-level physiological profiling approach, utilizing 96-well Biolog FF microplates, showed discernible variations in the utilization of 95 diverse carbon sources among the treatments. Notably, arbutin, l-arabinose, Tween 80, and succinamic acid demonstrated a strong positive association with *Mortierella*. Our findings demonstrate that a single application of mefenoxam at its recommended rate triggers substantial taxonomic and functional shifts in the soil fungal community. Considering this impact, the conventional agricultural practice of repeated mefenoxam application is likely to exert considerable shifts on the soil ecosystem that may affect agricultural sustainability.

## Introduction

Plant pathogenic fungi and fungal-like organisms (FLO), such as oomycetes are the major causes of preharvest and postharvest losses, accounting for a 20 to 40% reduction in crop yield^1,2^. Yield reductions due to infections by fungi and FLO result in a global food crisis and a massive economic loss estimated at over $100 billion^2–4^. Therefore, management of such plant diseases is necessary and fungicides have played a vital role in controlling the growth and survival of plant pathogenic fungi and FLO^1,2,4,5^. The global agricultural chemical pesticide market is anticipated to reach 122.1 billion in 2031 with an annual growth rate (CAGR) of 5.5%^5^. Over 7% of the global fungicide market targets single sites, making it easy for plant pathogens to develop resistance to them^6^. In addition, the frequent application of chemicals with a similar mode of action leads to a decline in their performance due to the development of resistance in the pathogen population^1,2^. Moreover, chemical fungicides are harmful to nontarget organisms and only a small amount (0.1%) of the applied fungicides reach their intended target, not to mention their persistence and bioaccumulation in the environment^1^.

Ridomil Gold SL (45.3% a.i. mefenoxam) is a widely used fungicide in US agriculture^7,8^. Ridomil Gold SL (hereafter referred to as mefenoxam) was introduced due to metalaxyl resistance in several oomycete pathogens^6,9,10^. Mefenoxam is a superactive granular fungicide that can systemically control soilborne diseases caused by oomycetes and water mold, including *Pythium*-damping off, *Pythium* root rot, *Pythium* leak, and carrot cavity spot^6,9–12^. Mefenoxam is widely used in different crops such as tomatoes, onions, melons, soybeans, peas, carrots, apples, leafy vegetables and cotton, ornamentals, and turf and lawn^12^. In carrot production mefenoxam is used to control cavity spot caused by *Pythium* species^13^. In spite of resistance to mefenoxam being reported in several plant pathogens, including *Phytophthora* and *Pythium* spp.^12,14–16^, it is still widely used in the US^17^.

The soil microbiome plays a crucial role in plant health and improves plant fitness and function under abiotic stress^18–21^. However, applications of chemical fungicides can lead to a long-term influence on soil microbes and soil ecosystem processes, such as nitrification and soil enzyme activities, which are essential for plant productivity^22–24^. Therefore, it is important to evaluate the impact of fungicides on nontarget organisms and the environment^25^. Previous studies suggested that the application of mefenoxam may have a deleterious effect on nontarget fungal populations^26,27^. However, details on its impact on the fungal community profile are lacking^26,27^. A study by Demanou et al.^26^ compared the number and position of the amplicon band, but failed to show the specific fungal taxa that were affected or enriched after mefenoxam application. Therefore, the objective of our study was to evaluate the effects of mefenoxam on the taxonomic and functional diversity of soil fungal communities in carrot cultivation. We hypothesized that the effects of mefenoxam on soil fungi would be minimal and temporal because it targets oomycetes and rapidly degrades in soil. In this study we applied the recommended dose of mefenoxam (0.24 ppm) to the soil in which carrots were grown. The experiment was performed both with and without the inoculation of *Pythium*, and the soil samples were collected after 2- and 12-weeks post mefenoxam application. The MiSeq raw sequence data were analyzed using QIIME2 and the predicted functional diversity was assessed using FUNGuild. Additionally, we examined the impact of mefenoxam on the metabolic activities of soil fungal communities using a Biolog FF MicroPlate. The results revealed that application of mefenoxam in the presence of *Pythium* led to significant, albeit temporary, alterations in fungal community assembly, ecological functions, and diverse carbon source utilization in carrot-cultivated soil.

## Material and methods

### Collection and organization of fungicide utilization data

**Figure 1:**
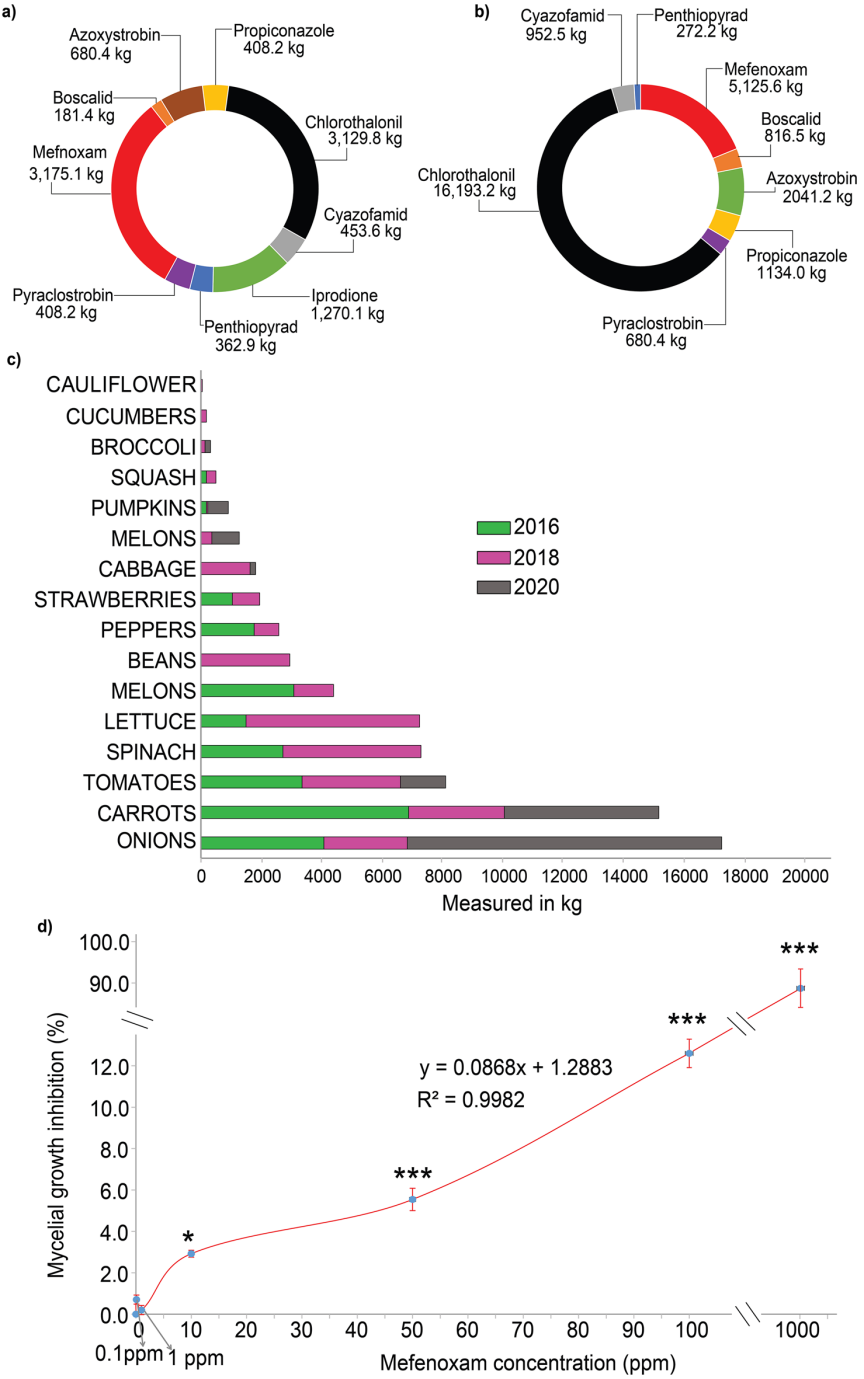
Chemical fungicide utilization and mefenoxam sensitivity assay: Pie charts showing chemical fungicides used in carrot production in California in (**a**) 2018 and (**b**) 2020; (**c**) a bar graph reporting mefenoxam usage in vegetable fields in the USA in 2016, 2018 and 2020; and (**d**) percent reduction in mycelia *Pythium irregulare* 24 h after being added to growth media amended with different concentrations of mefenoxam. Data are the mean (n = 5) ± standard error. Asterisks indicate statistically significant differences between the untreated control and mefenoxam concentrations, *(p < 0.05) and ***(p < 0.001).

The statistical data of fungicide utilization trends in carrot fields across the United States were obtained from publicly accessible USDA National Statistic Services (USDA-NASS)^8^. Furthermore, data on mefenoxam usage on various crops in California were analyzed. As some of the data were confidential or not available in the database, the data available since 2000 were combined and summarized as shown in this research (Fig. 1a and b, Table S1).

### Isolation and identification of *Pythium* species

*Pythium* species were isolated from cavity spot lesions of diseased carrots harvested from Kern County, California. The area surrounding the lesions was excised and disinfected with 10% bleach for 3 min, rinsed 3 times in sterile distilled water and air dried in a running biosafety cabinet. The tissue was cut into 5 mm pieces that were pushed into isolating media that consisted of 1% water agar with 10 µg ml^–1^ of carbendazim and pentachloronitrobenzene (PCNB) each and 50 µg ml^–1^ of ampicillin and rifampicin each^26^. The *Pythium* species were subcultured in 20% clarified V8 media with 1% agar. The isolates were identified by observing their morphology under a microscope and molecular technique using *Pythium*-specific primers^28,29^.

### In vitro mefenoxam sensitivity assay

To test the efficacy of mefenoxam against the three different *Pythium* species (*P. irregulare*, *P. ultimum* and *P. sulcatum*), 20% clarified V8 agar plates were amended with mefenoxam at different concentrations. Mefenoxam was dissolved in sterile water and added to autoclaved growth media that had been cooled to 65 °C in a waterbath. Media were thoroughly mixed and poured into Petri dishes. The final concentrations of mefenoxam tested were: 0, 0.1 ppm, 0.24 ppm, 1 ppm, 10 ppm, 50 ppm, 100 ppm and 1000 ppm. The efficacy tests were carried out by inoculating the growth media amended with mefenoxam with a 5 mm plug from an actively growing 2-day-old *Pythium* culture that was added to the center of each Petri dish and incubated at 25 °C in the dark for 48 h. Growth was calculated by measuring the diameter of the culture twice and averaging to obtain the values for each concentration. The experiment was laid out in a completely randomized design and replicated five times. Each treatment was repeated two times.

### Growing carrots in the greenhouse

Carrots, Crispy Cut variety, were seeded in 3 L pots (n = 20) using UC Soil Mix III (57% plaster sand, 43% peat moss with the addition of KNO_3_, 0.89 kg/m^3^ limestone flour, 0.74 kg/m^3^ phosphate, 2.22 kg/m^3^ dolomite, 41.5 g/m^3^ magnesium, 18 g/m^3^ manganese, 77.1 g/m^3^ iron, 30 g/m^3^ zinc, and 65 g/m^3^ copper). A PVC cylinder with a height of 15 cm and a diameter of 3.8 cm was placed in the center of each pot to allow a later *Pythium* inoculation without disruption of the carrot-root zone^30^. Carrots were inoculated with the *Pythium* isolate and treated with mefenoxam 28 days after planting. They were irrigated with a 1% solution of Peters Mix fertilizer (Peter’s 21-5-20 Excel Multi-Purpose, Scotts, USA) twice a week for the first month and placed on an automatic drip irrigation system thereafter. Each pot contained 4 carrots. Plants were harvested 16 weeks after sowing.

### *Pythium* inoculation and mefenoxam application

Vermiculite was autoclaved two times for one hour within 24 h^31^ and once more for 30 min before 0.5 L of 20% V8 broth was added to 1 L of the sterilized vermiculite. Ten 5 mm plugs from a 2-day-old *Pythium* culture were added to the vermiculite and incubated at 25 °C for 21 days with regular shaking by hand. The inoculum density was determined by diluting *Pythium* colonized vermiculite in 0.2% water agar and plating on 10% V8 agar plates amended with PARP (pimaricin 10 μg/ml, ampicillin 250 μg/ml, rifampicin 10 μg/ml, PCNB 25 μg/ml) according to Ref.^31^. To calculate the *Pythium* colony forming units per gram (cfu/g) of colonized vermiculite, 1 mL of the diluted *Pythium* suspension was plated onto V8 PARP media plates (n = 5), and total colonies were counted and averaged after 24 h. Three different *Pythium* species and 2 isolates per species were used in this experiment. Each of the six *Pythium* isolates contributed 667 cfu/g to the final inoculum density of 4000 cfu/g. The final inoculum density was achieved by diluting the vermiculite with steam-sterilized UC Mix III. To inoculate the plants, the PVC pipe in the middle of the pot was removed gently and the inoculum was added into its place. Each pot was inoculated with 200 g of inoculum adjusted to a final inoculum density of 4000 cfu/g. The control inoculum received the same amount of sterile vermiculite with 20% V8 broth, but it was not inoculated with *Pythium* spp.

Mefenoxam was applied to the greenhouse grown carrots at the recommended rate of 0.24 ppm. It was dissolved in water to the appropriate concentration and added to the soil to reach the final maximum recommended application rate described by the manufacturers. The fungicide was added to the soil the same day *Pythium* spp*.* were inoculated into the soil. The experimental pots were arranged in a completely randomized design. The experiments were replicated 5 times and repeated 2 times. Hence, the present study encompassed the following treatment groups: neither *Pythium* inoculation nor mefenoxam application (CKNP), *Pythium* inoculation without mefenoxam application (CKP), mefenoxam application without *Pythium* inoculation (RNP), and both *Pythium* inoculation and mefenoxam application (RP).

### Soil sample collection, DNA extraction and Illumina library preparation

Soil samples were collected at 2 weeks (T1) and 12 weeks (T2) after soil treatment with mefenoxam (Fig. S1a). Push cores with a 1 cm diameter were used to sample soil from a 3 cm depth. Total DNA was extracted from the soil samples and the Illumina sequencing library was prepared as described in our previous work^32^. Briefly, total environmental DNA was extracted from 0.25 g of soil samples using the DNeasy Powersoil kit (Qiagen, Valencia, CA, USA) following the manufacturer’s instructions. DNA quality was checked using an Implen NanoPhotometer (Implen, Westlake Village, CA, USA). To characterize the fungal community, amplicon libraries of the fungal internal transcribed spacer 2 (ITS2) region were amplified using universal fungal primers 5.8SFun and ITS4Fun^33^. A two-step PCR dual indexing inline barcoding procedure and primers were used to generate amplicons for Illumina sequencing^33,34^. Phusion high-fidelity PCR master mix with HF buffer (Thermo Scientific) and 0.2 µM primers were used as PCR reagents with 1 µL of extracted DNA for the template. PCRs were carried out on the Bio-Rad T100 thermal cycler with the initial denaturation at 98 °C for 30 s, followed by 43 cycles of 98 °C for 10 s, 55 °C for 40 s and 72 °C for 2 min, and a final extension at 72 °C for 10 min adapted from Kembel and colleagues^35^. PCRs were screened for quality and fragment size using gel electrophoresis with a 1% agarose gel. Amplicons from successful PCRs were purified using the Agencourt AMPure XP beads protocol (Beckman Coulter, Brea, CA, USA), except that SPRI beads (Beckman Coulter, Brea, CA, USA) were used and all ethanol washes were performed using 80% ethanol. Cleaned DNA products were used as a template in a second PCR under similar conditions as described above except 0.3 µM primers were used^34,35^ and 7 cycles were used with an annealing temperature of 65 °C. PCRs were screened as described for the initial PCR. DNA concentrations were measured using Qubit 2.0 Fluorometer (Life Technologies, Carlsbad, CA, USA), and amplicons were pooled in equal molar concentrations of 5 nM for sequencing. The samples were submitted to the UC Riverside Genomics Core Facility where library quality was assessed using a 2100 Bioanalyzer (Agilent), and the libraries were sequenced using a MiSeq sequencer (Illumina) and MiSeq Reagent Kit version 3 (Illumina) with 2 × 150 cycles.

### Community level physiological profiling of fungal communities

The changes in the metabolic activities of soil fungal communities in response to mefenoxam and/or *Pythium* inoculation were examined using a Biolog FF MicroPlate. The Biolog FF Microplate is a 96-well plate that contains 95 distinct classes of carbon sources that are used to assess the functional capabilities of the diverse fungal communities (Biolog, Inc., Hayward, California, USA). To assess changes in the metabolic activities due to treatments, a 1 g fresh soil sample was suspended in 99 ml of sterile NaCl solution (9 g L^–1^), vortexed for 20 min at room temperature, and kanamycin was added at 100 µg ml^–1^ to suppress bacterial growth while favoring fungal growth^36^. Subsequently, aliquots (100 µl) of the suspension were added into each well of the microplate and incubated at 27 °C for 6 days^37,38^. Absorbance readings were taken at 490 nm every day for six days using a SpectraMax iD5 Multi-Mode microplate reader. The experiment was replicated three times. Negative values were converted to zero. Average well color development (AWCD) was calculated according to the method followed by Ref.^39^. The differences in carbon-sources utilization by soil fungal communities in different treatments over different timepoints were determined based on principal component analysis. For analysis carbon sources were categorized into carbohydrates, amino acids, amines and amides, carboxylic acids, polymers and miscellaneous^40^.

### Bioinformatics analysis

ITS2 datasets were analyzed following the QIIME2 analysis workflow (version 2022.8)^41^. Briefly, raw paired-end reads (300 bp for ITS2) were demultiplexed and barcodes trimmed using cutadapt (v4.1) with parameters `-e 1 –discard-untrimmed^42^. Demultiplexed reads were imported into QIIME2 for subsequent analysis. Imported sequences were subjected to denoising and clustering analysis using DADA2^43^. For the ITS2 dataset, sequence data were first downloaded from the UNITE database^44^ for QIIME, and sequences at the 99% similarity level were trained using the QIIME2 'fit-classifier-naive-bayes' module. Classification was assigned from the newly trained classifiers. Mitochondria and chloroplast reads were also filtered and removed after the initial taxonomic classification. The number of sequence reads for all samples was rarefied to an equal sampling depth of 80,823 reads per sample (Fig. S1b). The final normalized fungal microbiome dataset contained 522 amplicon sequence variants (ASVs) and taxonomy data were exported using QIIME2 and imported into R (version 4.1.3) for data processing, analysis and visualization.

The Fungal Functional Guild (FunGuild) database^45^, a python-written algorithm constructed with numerous different modules, was used to assign each fungal ASV into its ecological functional group. By taking into account all confidence rankings, assignments of ASVs into trophic modes and guilds were made. ASVs were classified as unassigned if there was no FunGuild database match.

Python (Python version 3.12.2)^46^, AWK^47^ and R (R version 4.2.3 with DECIPHER package)^48^ programs were employed to design species-specific primers for *Mortierella alpina*. Thirty-one genome reference sequences of *Mortierella* from NCBI were downloaded, and a custom database was curated for BLAST searches. Among the genome reference sequences, *M. alpina* strain CGMCC 20,262 was chosen, and its sequences were truncated into 300 bp fragments. These fragments were blasted using BLASTN to pinpoint unique regions specific to *M. alpina* within a customized database using the following parameters: -ungapped -max_target_seqs 300 -dust no -soft_masking false -qcov_hsp_perc 100 -max_hsps 1. Sequence fragments with low similarity were selected from the search results using the AWK programming language^47^. The chosen fragments were blasted using NCBI-BLAST search, and those with high similarity hits were chosen for further investigation. Sequence alignment and identification of unique primers specific to *M. alpina* were performed using the R program (R version 4.2.3) with DECIPHER package^48^. The primer’s specificity was tested through both in silico and in vitro assays. The in-silico PCR results demonstrated that the primer successfully amplified *Mortierella*, particularly, *M. alpina* without any gaps (data not shown). In vitro PCR also revealed that the primer pairs were highly specific to *M. alpina* strains AD021 and AD072, whereas related species like *Mortierella polycephala* KOD948, *Mortierella* sp GBAus30 and different genera such as *Linnemannia elongata* and *P. irregulare* were not amplified indicating that the primer pairs (Table S2) exhibited high specificity for *M. alpina* (Fig. S2).

### Statistical data analysis

The R program software (R version 4.2.3) was used for all downstream statistical data processing and visualization using different R packages, including dplyr, phyloseq^49^, vegan^50^, ggplot^51^ and complexHeatmap^52^. Homogeneity of variance and normality assumptions were tested using Levene test, Shapiro‒Wilk test and homogeneity of multivariate dispersion (PERMDISP) in R^53^. To increase the data normalization, fungal composition data were transformed using the centered log-ratio (clr)^54^. The statistical significant differences between treatments based on fungal composition, alpha diversity indices and CLPP data were compared using the agricolae R package^55^. The overall fungal community composition difference among treatments was determined using permutational multivariate analysis of variance (PERMANOVA) based on the weighted UniFrac distances^56^. Two-way PERMANOVA was performed to analyze the impacts of sampling time, treatments, and their interactions on fungal community structure. The basic codes for R, Python, and AWK used for primer design and data visualizations are available on GitHub at https://github.com/setubazie.

### Ethics approval and consent to participate

We confirm that all the methods were carried out in accordance with relevant institutional guidelines and regulations.

## Results

### Fungicide usage in the United States between 2000 and 2020

We curated data on the different chemical fungicides used in carrot fields in the United States between 2000 and 2020. According to the USDA-NASS, the chemical fungicides reported to have been used in carrot production in this time period are azoxystrobin, boscalid, chlorothalonil, copper hydroxide, mefenoxam, pyraclostrobin, sulfur, penthiopyrad and propiconazole (Table S1)^8^. The most frequently used fungicides in these 20 years were chlorothalonil and mefenoxam followed by azoxystrobin (Table S1). In California, mefenoxam was the second most utilized fungicide in carrot fields in 2018 and 2020 (Fig. 1a and b). According to USDA-NASS, several vegetable crops were treated with mefenoxam in 2016, 2018 and 2020 (Fig. 1c). The largest portion of mefenoxam applied in 2020 was on onion fields (54.9%), followed by carrots (27%) (Fig. 1c). California is the leading carrot producer in the United States, producing an average of 85% of the fresh carrots in the country. In carrot production, mefenoxam is used to control carrot cavity spot (CCS) caused by *Pythium* species, downy mildews and diseases caused by *Phytophthora* species. CCS is the leading fungal disease of carrot production in California. Although the exact amount of mefenoxam used in each state is not available, these results show that a significant amount of mefenoxam enters the soil each year in several vegetable-growing regions in the country.

### Mefenoxam sensitivity assay

In our greenhouse pot experiment, mefenoxam did not show significant reduction in CCS compared to the control (data not shown). In the in vitro assay, the maximum recommended concentration of mefenoxam, 0.24 ppm, did not reduce the mycelial growth of *P. irregulare* but suppressed growth of *P. sulcatum* and *P. ultimum* isolates (data not shown)*.* Therefore, we tested the sensitivity of *P. irregulare* mycelia to a range of concentrations of mefenoxam in vitro (Fig. 1d). The growth of the *P. irregulare* was significantly (p < 0.05) lower than that of the untreated control at 10 ppm, 50 ppm, 100 ppm and 1000 ppm of mefenoxam, but not at 1 ppm (Fig. 1d). At 100 ppm mycelial growth was reduced by 11.4% (Fig. 1d). Therefore, the isolate was labeled as resistant^57^.

### Temporal dynamics of soil fungal diversity

**Figure 2:**
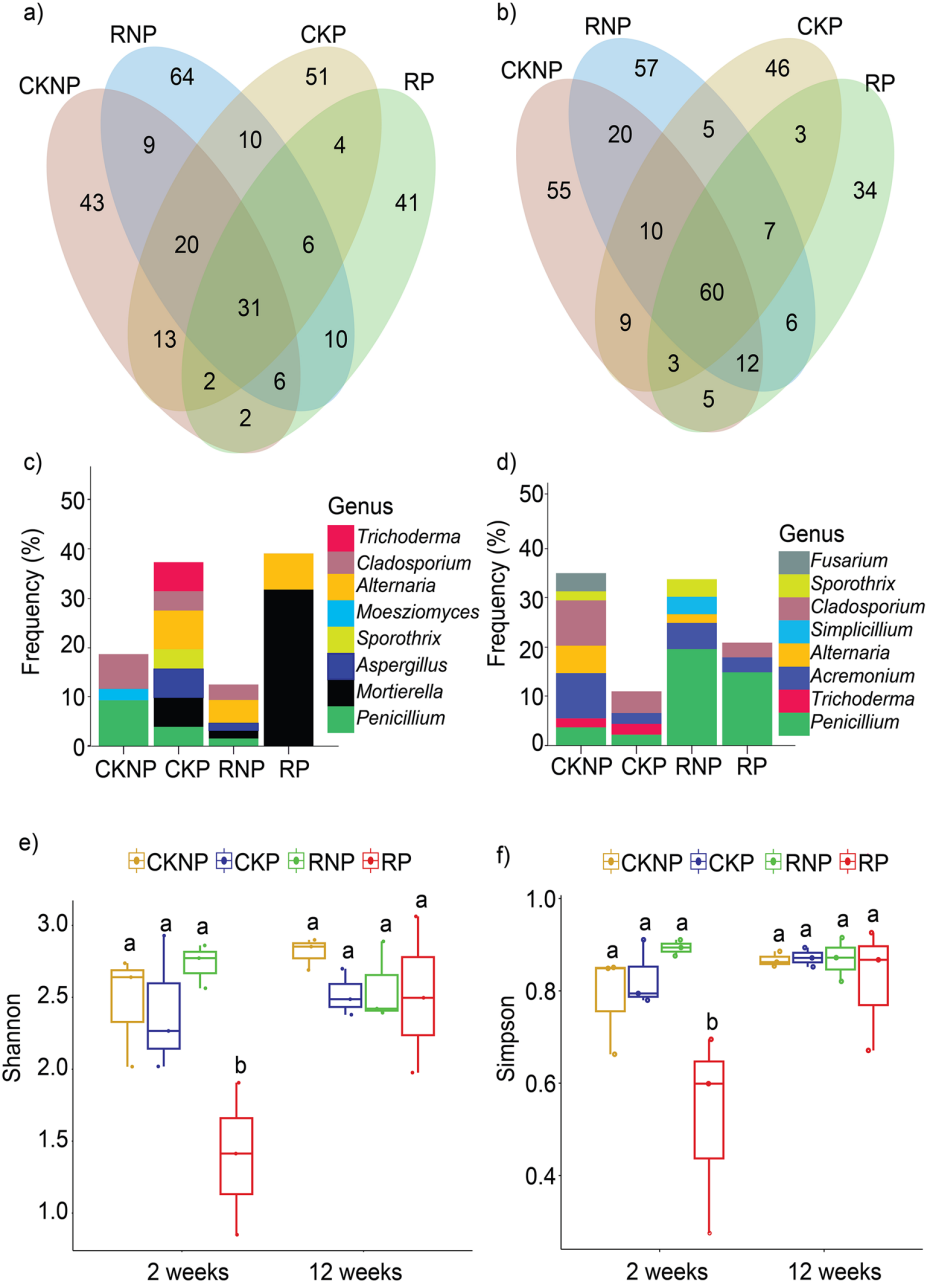
Venn diagrams and alpha diversity indices at two and twelve weeks after treatment: Venn diagram shows the unique and shared ASVs among treatments at T1 (**a**) and T2 (**b**). The unique ASVs at the genus level for each treatment at T1 (**c**) and T2 (**d**). Alpha diversity indices: Shannon (**e**) and Simpson (**f**) at T1 and T2. Different letters on the bars denote statistically significant differences between treatments at p < 0.05. T1 and T2 represent sampling times of 2 and 12 weeks, respectively. CKNP represents the control without *Pythium* inoculation or mefenoxam application, CKP represents *Pythium* inoculation without mefenoxam application, RNP represents mefenoxam application without *Pythium* inoculation, and RP represents *Pythium* inoculation and mefenoxam application.

We obtained a total of 16.7 × 10^6^ amplicon sequence reads across all samples with sequence numbers ranging from 80,823 to 454,594. The reads were rarefied to a minimum equal sequencing depth of 80,823 per sample (Fig. S1b), and 18 ASVs were excluded. The remaining 504 ASVs were used for analysis. Between sampling times T1 and T2, the number of ASVs unique to the CKNP soil samples increased by 12. However, the number of ASVs unique to CKP, RNP, and RP declined by 5, 7 and 7, respectively (Fig. 2a,b). Interestingly, the number of ASVs shared among all treatments increased from 31 at T1 to 60 at T2 (Fig. 2a,b), suggesting that the fungal communities in all the treatments were more similar at T2 than at T1. Analysis of the unique ASVs within each treatment showed that the majority of the 41 unique ASVs in RP samples at T1 were identified as *Mortierell*a (Fig. 2c). This suggests an enrichment of *Mortierella* due to RP treatment. On the other hand, at T1, the unique ASVs in the CKNP were predominantly *Penicillium* and *Cladosporium*, while CKP’s unique ASVs were largely dominated by *Aspergillus* and *Alternaria* (Fig. 2c). At T2, *Acremonium* ASVs were found in all samples, while *Trichoderma* was observed only in samples that were not treated with mefenoxam (CKNP and CKP) (Fig. 2d). In addition, the unique ASVs of RNP and RP were dominated by *Penicillium* at T2 (Fig. 2d).

**Figure 3:**
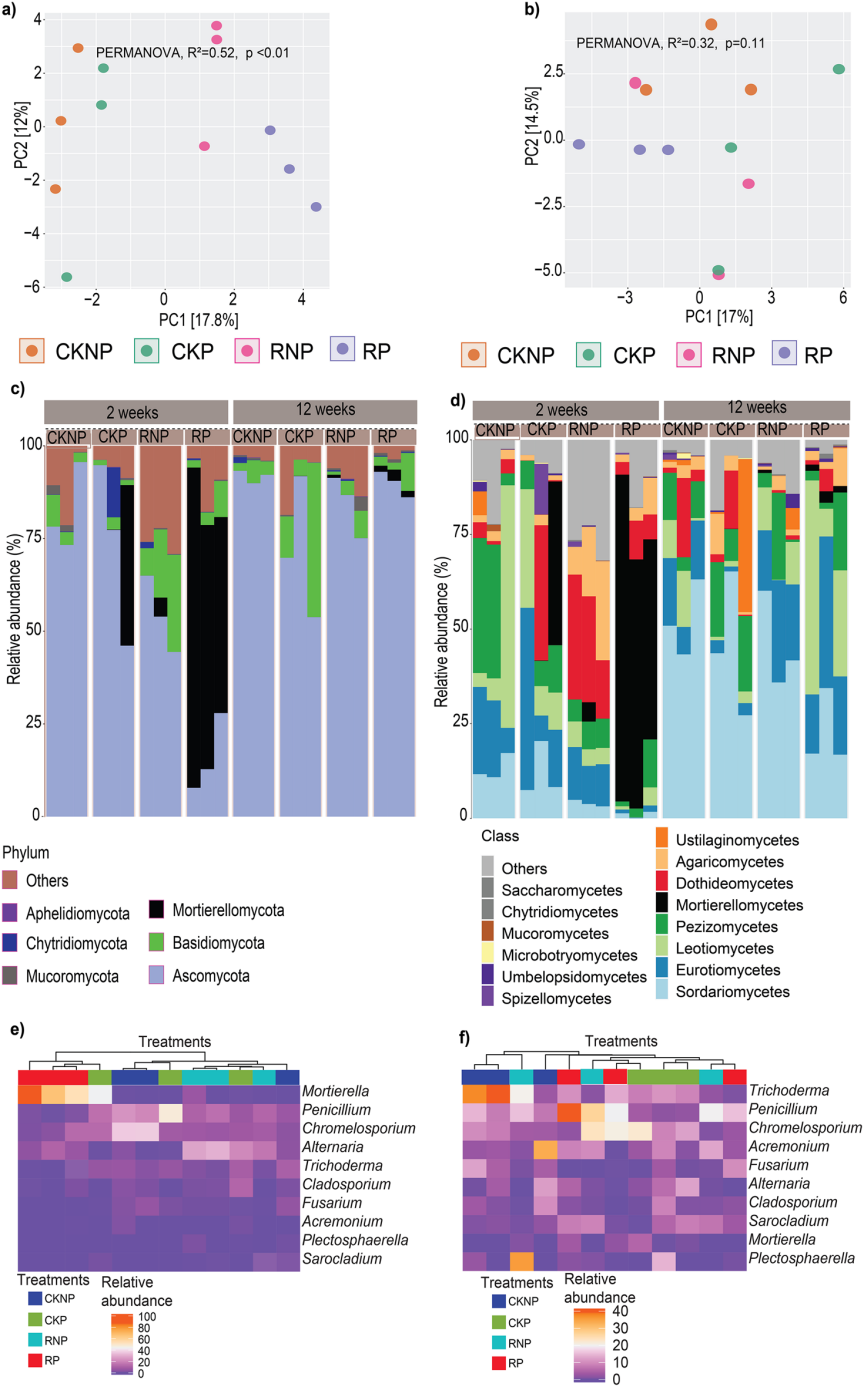
Ordination and composition. Principal component analysis plot showing beta diversity, measured based on UniFrac distances, at T1 (**a**) and T2 (**b**). Relative abundance of fungal phyla (**c**) and classes (**d**) in different treatments at T1 and T2. Heatmap showing the relative abundance of the top 10 genera in different treatments at T1 (**e**) and T2 (**f**). T1 and T2 represent sampling times 2 and 12 weeks after treatment respectively. CKNP represents the control without *Pythium* inoculation or mefenoxam application, CKP represents *Pythium* inoculation without mefenoxam application, RNP represents mefenoxam application without *Pythium* inoculation, and RP represents *Pythium* inoculation and mefenoxam application.

In addition, our data indicated that treatments had a significant (p < 0.05) effect on fungal alpha diversity at T1, as indicated by the Shannon and Simpson indices (Fig. 2e,f; Table S3). At T1, RP had the least fungal alpha diversity and was significantly different from all the other treatments (Fig. 2e). However, at T2, the alpha diversities of all treatments showed no significant difference from each other (Fig. 2f). Beta diversity analysis at T1 showed that the fungal community structure was significantly different among treatments (PERMANOVA R^2^ = 0.52; p < 0.01) (Fig. 3a). The fungal community structure in RP soil samples were clustered closely together, distinct from the other treatments. In spite of the differences observed at T1, the community structure across different treatments was not significantly different at T2 (PERMANOVA R^2^ = 0.32; p = 0.11) (Fig. 3b). Similarly, there were substantial compositional variations in the fungal taxonomy among treatments at T1 but not at T2 (Fig. 3c–f), indicating that the fungal community in RP would be gradually restored to that of the control soil.

### Effects of treatments on fungal community composition

The most abundant fungal phylum across all treatments was Ascomycota. Ascomycota comprised over 70% of the relative abundance in all samples at both time points, except for mefenoxam-treated samples at T1 (Fig. 3c, Table S4). ANOVA revealed significant (P < 0.05) interaction effects between treatments and sampling time for Ascomycota, Mortierellomycota and Basidiomycota (Table S4). At T1, the abundance of Ascomycota was highest in CKNP but significantly reduced in RP. However, the opposite pattern was observed for Mortierellomycota and Basidiomycota, which increased in RP and RNP, respectively, compared to CKNP (Fig. 3c, Table S4). Despite these significant changes at T1, the relative abundance of Basidiomycota was reestablished to levels found in CKNP after 12 weeks (Fig. 3d, Table S4). For Chytridiomycota and Mucoromycota, there was no statistically significant (P > 0.05) change in their relative abundance across all treatments at both time points (Table S4).

At the class level, both treatments and sampling time showed significant (p < 0.05) effects on the relative abundance of Sordariomycetes and Mortierellomycetes (Fig. 3d, Table S5). The relative abundance of Sordariomycetes was highly reduced in mefenoxam-treated soil (RNP and RP) when compared to the mefenoxam-untreated samples (CKNP and CKP) at T1, indicating the effect of mefenoxam on these fungal classes. Interestingly, ANOVA revealed a significant interaction effect between treatments and sampling time (P < 0.05) on the relative abundance of Eurotiomycetes and Agaricomycetes (Table S5). While RP significantly reduced the relative abundance of Eurotiomycetes at T1, their abundance increased to levels found in the RNP at T2 (Fig. 3d, Table S5). The relative abundance of Agaricomycetes increased significantly in RNP compared to the other treatments at T1; however, this increase was not observed at T2. Despite ASVs being assigned to the fungal classes Leotiomycetes, Pezizomycetes, Dothideomycetes, Ustilaginomycetes, Spizellomycetes, Umbelopsidomycetes, Microbotryomycetes, Mucoromycetes, Chytridiomycetes and Saccharomycetes, no significant changes were observed between treatments and sampling times (Table S5).

The five most abundant fungal genera in all the treatments and at both time points were *Penicillium*, *Mortierella, Chromelosporium, Trichoderma and Alternaria* (Fig. 3e,f, Table S6). *Penicillium* was found to be negatively impacted by RP at T1, but its relative abundance increased at T2 (Fig. 3e,f). *Mortierella* was found to be highly abundant in RP at T1. The ASVs classified as *Mortierella* exhibited a notable highly similarity with *M. alpina,* as indicated by the results of the BLAST search. Subsequent PCR assay using primers specific to *M. alpina* confirmed a distinct and strong band in the RP samples, whereas no band was observed in the other treatments (Fig. S2). However, RP and RNP significantly decreased the relative abundance of *Trichoderma* and RNP significantly increased the relative abundance of *Alternaria* at T1 but not at T2 (Table S6). Indicating that treatment with mefenoxam alone (in absence of *Pythium*) significantly increased the relative abundance of *Alternaria* for a short time. In addition, *Fusarium* species were abundant in CKNP at T1 but less abundant in other treatments. The genera *Acremonium*, *Sarocladium*, and *Plectosphaerella* were more abundant at T2 than at T1 (Fig. 3e,f, Table S6).

### Changes in fungal ecological function in response to treatments

**Figure 4:**
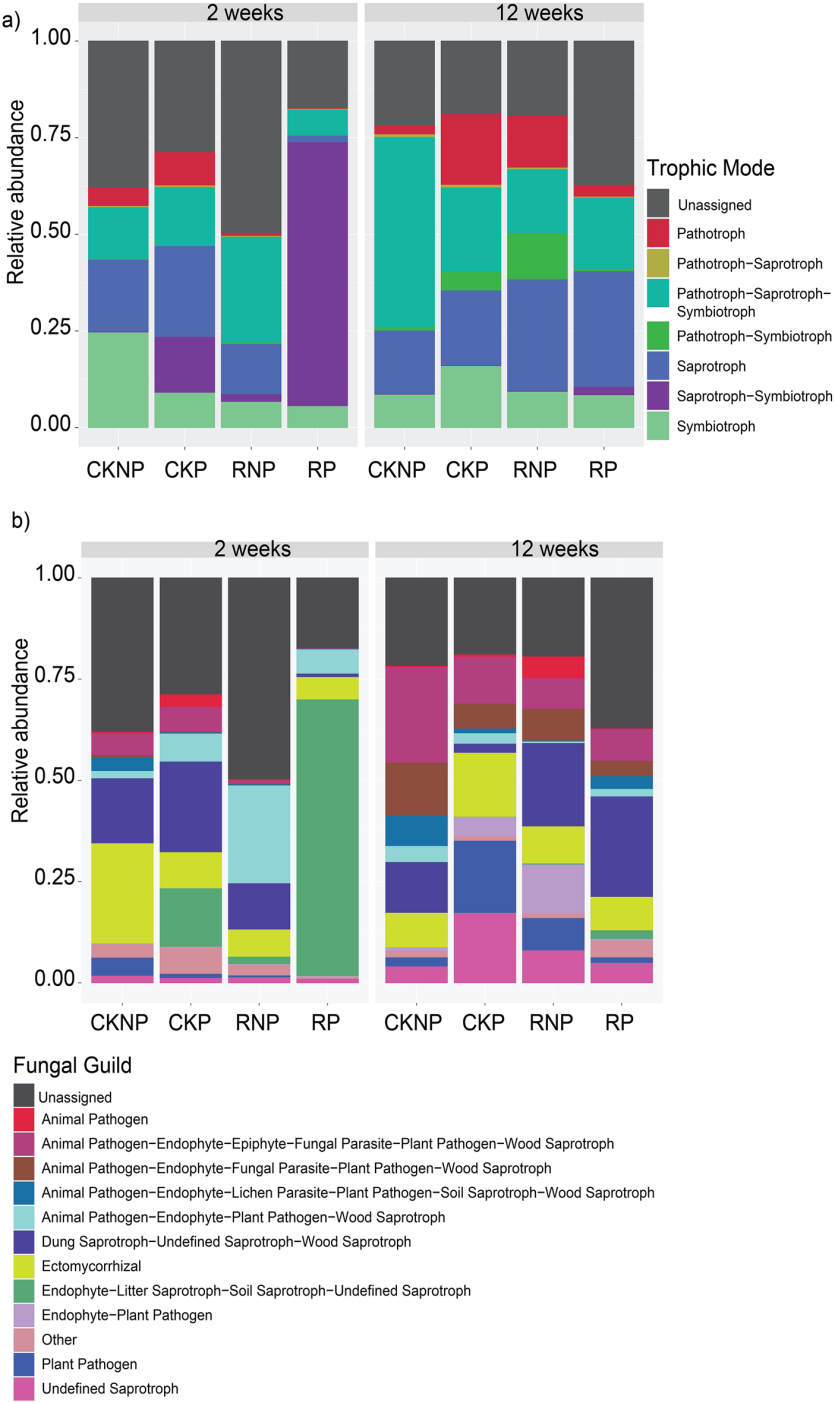
Predicted ecological functions of fungal communities under different treatments. The relative abundance of ASV richness assigned to each of the fungal trophic modes (**a**) and ecological guilds (**b**) at 2 and 12 weeks after application. CKNP represents the control without *Pythium* inoculation or mefenoxam application, CKP represents *Pythium* inoculation without mefenoxam application, RNP represents mefenoxam application without *Pythium* inoculation, and RP represents *Pythium* inoculation and mefenoxam application.

The ecological roles of fungal communities were predicted using the FunGuild database at broadly defined trophic modes and guilds (Fig. 4a,b). Over 50% of the ASV taxa had predicted functions while the remaining taxa were unassigned. Our analysis showed that the treatments had the potential to disrupt the ecological function of soil fungal communities. The predicted functions in RP at T1 were dominated by the saprotroph-symbiotroph trophic level, but the predicted abundance of symbiotrophs was only approximately half of that in CKNP. Moreover, the predicted relative abundances of symbiotrophs and pathotrophs were low in mefenoxam treated soil (RNP and RP) compared to CKNP at T1, but not at T2 (Fig. 4a). At T2, the predicted relative abundance of saprotrophs was higher in all mefenoxam-treated samples (RNP and RP), whereas CKNP showed a higher abundance of pathotroph-saprotroph-symbiotrophs (Fig. 4a). Fungal guild analysis also showed that the RP treatment reduced the dung -wood saprotroph, and plant pathogen functional groupings at T1 compared to CKNP, although such a reduction was not found at T2 (Fig. 4b). In contrast, the RP showed a higher predicted abundance of endophytic litter saprotrophs at T1 (Fig. 4b). These findings imply that the application of mefenoxam and *Pythium* inoculation played a role in driving these predicted ecological functional changes.

### Community level physiological profiling (CLPP) using biolog FF plates

**Figure 5:**
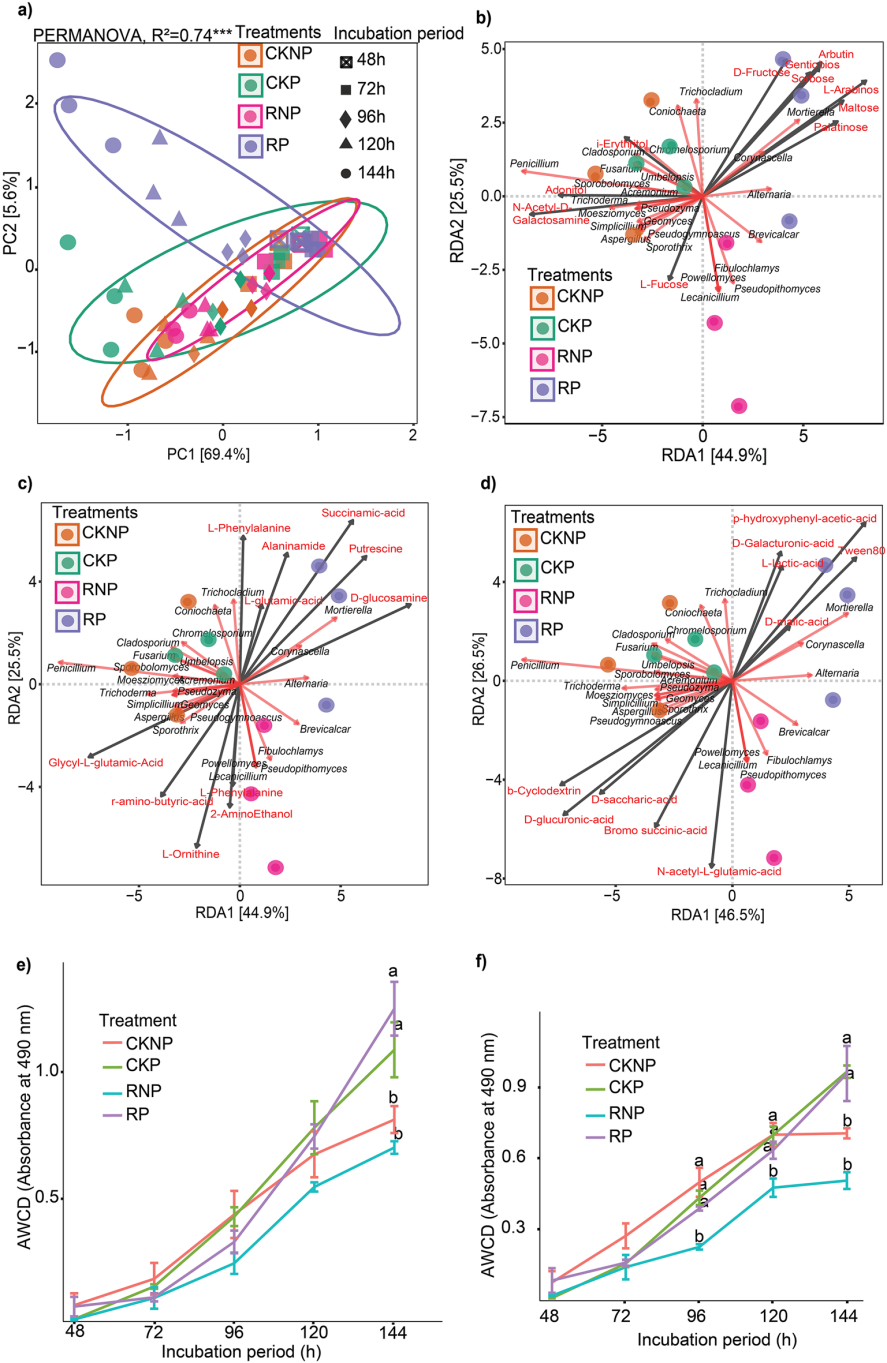
Community level physiological profiling (CLPP) showing alterations in the functional diversity of fungal communities using Biolog FF plates. (**a**) Principal component analysis exhibiting the utilization of different carbon sources by soil fungal communities in different soil treatments. Distance-based redundancy analysis showing the relationship between fungal communities and carbon sources: (**b**) carbohydrate, (**c**) carboxylic acids and (**d**) polymers. Utilization of different carbon-source categories by treatments based on AWCD: (**e**) carbohydrate groups, (**f**) carboxylic acid groups. CKNP represents the control without *Pythium* inoculation or mefenoxam application, CKP represents *Pythium* inoculation without mefenoxam application, RNP represents mefenoxam application without *Pythium* inoculation, and RP represents *Pythium* inoculation and mefenoxam application.

**Figure 6:**
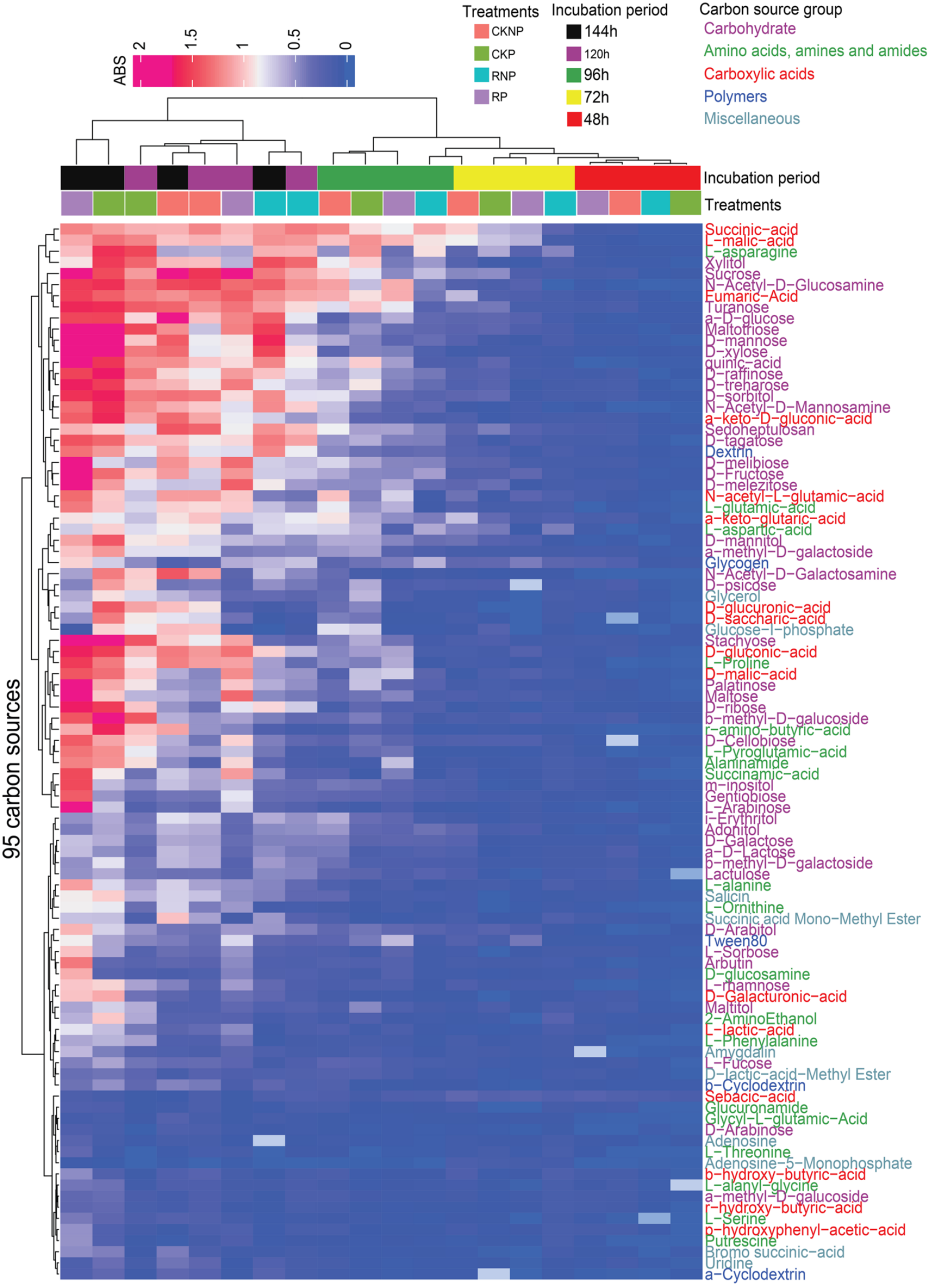
Heatmap showing carbon utilization of 95 different carbon sources of the Biolog FF plates. The data shown are based on absorbance at 490 nm. CKNP represents the control without *Pythium* inoculation or mefenoxam application, CKP represents *Pythium* inoculation without mefenoxam application, RNP represents mefenoxam application without *Pythium* inoculation, and RP represents *Pythium* inoculation and mefenoxam application.

In the present study, alterations in the functional diversity of fungal communities were investigated using the community level physiological profiling (CLPP) approach. The 96-well Biolog FF microplates containing 95 distinct carbon sources, alongside a non-carbon source were employed. The results showed discernible variations in the utilization of diverse carbon sources among the treatments (Fig. 5a). RP samples exhibited high utilization of arbutin, L-arabinose and Tween 80 (Figs. 5b, 6, S3) compared to the other treatments. Simultaneously, RP showed the lowest utilization of N-acetyl-D-galactosamine (Figs. 6, S3) and glycyl-L-glutamic acid (Figs. 6, S3). Notably, carbohydrate carbon sources such as D-fructose, gentiobiose, sorbose, maltose, palatinose, arbutin, and l-arabinose demonstrated a strong positive association with *Mortierella* and RP samples (Fig. 5b). Conversely, CKNP showed the highest utilization of N-acetyl-D-galactosamine (Fig. 5b) and glycyl-L-glutamic acid (Fig. 5c) carbon sources, which showed a strong positive link with *Penicillium*. Tween 80 from the polymer group also had a positive relationship with *Mortierella* and RP samples (Fig. 5d).

The AWCD results indicated a lack of significant variation among treatments during the initial 120 h of incubation for most carbon sources, except for carboxylic acid which had significant differences starting at 96 h (Figs. 5e,f, 6, S3). At 144 h, an overall increased metabolism of carbon sources was noted (Figs. 5e,f, 6, S3). At 144 h, the treatments exhibited statistically significant (p < 0.05) differences based on AWCD. RP and CKP (both of which were inoculated with *Pythium*) demonstrated the highest utilization (Fig. 5e,f), with the exception of polymers where insignificant differences were observed (Fig. S3). Overall, carbohydrate carbon sources exhibited comparatively robust utilization across samples (Fig. 5e). Notably, N-acetyl-d-glucosamine, α-d-glucose, d-xylose, and d-sorbitol were the most highly utilized individual substrates across the treatments at the end of the incubation time. In contrast, l-threonine, adenosine-5-monophosphate, glucuronamide, and d-arabinose were the least metabolized substrates across the treatments (Fig. 6).

### Effect of mefenoxam on fungal diversity in soil without carrot cultivation

The impact of mefenoxam was further investigated in soil without any carrots growing in it, referred to as bare soil. The results revealed that the fungal community structure in the bare soil was altered by the treatments at both time points, T1 and T2, primarily due to RP (Fig. S4a,b). PCA plots of the fungal community structure showed that RP and RNP communities were distinct from CKNP and exhibited tighter clustering at T2 (Fig. S4a–d). In carrot-grown soil, the fungal community structure of RP was not statistically significantly different from that of CKNP at both T1 and T2. However, in the bare soil, there was a significant difference at T2 (Fig. S4c,d). As observed in the carrot-cultivated soil, RP enriched Mortierellomycota in bare soil more at T1 than at T2 (Fig. S4e,f). However, the relative abundance of Mortierellomycota was higher in soil with carrots than in bare soil. CKNP in carrot cultivated soil exhibited a significantly (p < 0.05) higher alpha diversity compared to the bare soil at T2 (Fig. S4g,h). While RP had a significant effect on the alpha diversity of carrot-cultivated soil at T1, this effect was not observed at T2. In contrast, in bare soil, the pattern was reversed, with the alpha diversity of RP being slightly higher than that of CKNP at T2, but not at T1. This could be attributed to the impact of carrots.

## Discussion

In this study, we investigated the impact of mefenoxam, one of the frequently used fungicides in the USA, on the soil fungal communities during carrot cultivation. Our data indicate that mefenoxam may not only fail to control the targeted plant pathogen due to the development of resistant strains but also have adverse effects on the nontarget soil fungal community. Although the effects of mefenoxam on soil fungi have been speculated, this is the first study, to our knowledge, that clearly demonstrates its ability to significantly reduce soil fungal diversity. Furthermore, it sheds light on its impacts on beneficial soil fungi, and its possible detrimental effects on soil health and agricultural productivity.

Based on the data obtained from the USDA-NASS, the fungicides most frequently used in significant amounts are chlorothalonil and mefenoxam^8^. Chlorothalonil, a nonsystemic protectant fungicide, is widely used in the USA^58^. Previous studies have documented notable effects of chlorothalonil on soil fungal community structure and function, signifying potential threat to the agroecosystem^22,59^. Mefenoxam, a systemic fungicide, controls oomycete diseases, including *Pythium* infections in carrots^13,60^. However, its repeated application as a foliar spray, seed treatment, and soil treatment results in the emergence of resistant *Pythium* isolates^14,61^. Mefenoxam resistance has been documented previously in California^57,62^, and the threshold for *Pythium* resistance has been set at less than 60% mycelial growth reduction at 100 ppm of mefenoxam^63^. Therefore, the *P. irregulare* isolate we used demonstrated resistance to mefenoxam, as it showed only an 11% reduction in mycelial growth at 100 ppm mefenoxam. Despite the presence of mefenoxam-resistant *Pythium* isolates in California, mefenoxam continues to be one of the widely used chemical fungicides for the management of CCS.

Numerous studies have shown that mefenoxam has detrimental effects on soil ergosterol and dehydrogenase^16,25,27,64^, which correlate strongly with fungal biomass^65^. Notably, while the potential impact of mefenoxam on the fungal community has long been speculated based on amplicon band profile comparisons^66^, the precise impact on the fungal community profile has remained undetermined^27,66^. Our findings indicated that a single application of mefenoxam exerted a temporary yet considerable impact on the taxonomic and functional diversity of the fungal communities. Such shifts could have deleterious effects on soil functions and crop productivity, as fungi play crucial roles through their array of extracellular enzymes that facilitate diverse ecosystem processes^67^. These functions include the breakdown of different organic matter sources, thereby regulating the carbon-to-nutrient balance^18,20,67–69^. Furthermore, changes in this balance between carbon mineralization and stabilization, may trigger additional consequences, including influencing the net flux of greenhouse gasses^69,70^. The resistance of one of the *P. irregulare* isolates used in the study to mefenoxam may have also contributed to the observed impact of RP treatment on fungal diversity, but further investigation is needed to confirm this. In addition, since Ridomil Gold SL contains 45.3% a.i. mefenoxam, the potential impacts of the other ingredients (54.7%) on fungal diversity need to be studied.

We found a very low predicted symbiotroph function in the RP compared to CKNP. Reduction of symbiotic associations in the soil can be detrimental to productivity^67,71^ as they enhance plant nutrient absorption and improve plant performance and fitness^72–75^. The impact of fungicides on nontarget fungal communities has been documented in previous studies^76–78^. However, our study stands as the first report on mefenoxam’s effect on both the taxonomic and functional diversity of fungi. Given that, in our experiment, mefenoxam was applied only once during the cropping season, while in agricultural production it may be applied multiple times in a single season, the effects on the soil fungal taxonomic and functional diversity could potentially be more drastic than observed in our study^79,80^. Furthermore, our study highlights the fact that carrot cultivation itself impacts soil fungal community composition and diversity, indicating the need for further investigation. This concurs with the finding of Noel et al. that reported that the impact of fungicides on microbiomes is dependent on crop management practices^77^.

The lower alpha diversity in the RP within 2 weeks likely resulted in reduced fungal competition, allowing *Mortierella* to flourish. Similarly, previous findings noted that reduced competition can promote the flourishing of certain keystone taxa in pesticide-contaminated areas^81^. Similarly, in a study by Zhao et al.^82^, *Mortierella* was found to dominate the vacant niches following the application of the broad-spectrum Dazomet. In addition, *Mortierella’s* adaptability to diverse environments^83^ and its capacity to degrade herbicides and pesticides^84–87^, may have enabled it to withstand disruptions induced by RP treatment. Interestingly, *Mortierella* species have been shown to be beneficial to agriculture^88,89^. However, *Mortierella* has also been linked to crop productivity decline^88^. Thus, further investigation is necessary to verify the potential influence of increased *Mortierella* abundance incited by RP on soil and plant health. It was intriguing to observe that application of mefenoxam alone (RNP) significantly increased *Alternaria* spp. *Alternaria* infections cause significant losses in carrot production in some parts of California (personal communication). Therefore, further investigation into this likely association between mefenoxam application and increased *Alternaria* spp. is necessary, given that mefenoxam is one of the key fungicides in conventional carrot farming. On the other hand, the RNP and RP treatments led to a decrease in the relative abundances of *Trichoderma* albeit temporarily. *Trichoderma* species have several uses in agriculture, such as controlling soilborne plant pathogens and insect pests and contributing to soil bioremediation^90,91^. Previous studies have documented the negative impacts of fungicides, including mancozeb, on the sporulation of *Trichoderma*^92^. However, there are reports of *Trichoderma* compatibility with fungicides for effective plant disease control^91^. The variable impacts of fungicides on nontarget microorganisms in different studies may depend on factors such as the nature of fungicides, dosage and the composition of soil microbiome^77^. This highlights the significant need for further investigation on effects of fungicides on the plant and soil microbiomes, and monitoring of fungicide use to minimize the risks to nontarget soil inhabitants and the emergence of resistant soilborne pathogens.

The differences in the metabolism of various carbon sources among the treatments observed in our study may be attributed to changes in the metabolic activities of fungal communities^38^. Consistent with this, our CLPP approach, utilizing 95 distinct carbon sources in 96-well Biolog FF microplates, has been previously employed to assess shifts in functional diversity^38,40^. Interestingly, despite the significantly lower taxonomic diversity observed in the RP treatment at T1, the overall metabolism of carbon sources in RP was found to be higher compared to CKNP, which exhibited higher taxonomic diversity. This observation may be attributed to the versatility of certain taxa, capable of performing multiple tasks effectively, and to the fact that the lower diversity could potentially enhance their performance due to reduced competition^93^. Furthermore, the specialized metabolic activities of *Mortierella* on fructose metabolism^94^ align with our results of high D-fructose utilization in *Mortierella*-dominated RP treatment. The strong positive association of Tween 80 with *Mortierella in* our results is supported by previous studies which indicated that *Mortierella* efficiently utilizes Tween 80, and its activity increased exponentially upon the addition of Tween 80^95^. In addition, the higher utilization of carbohydrates compared to polymers may be attributed to the fact that carbohydrates are easier to metabolize, because degradation of polymers by microbes requires more energy than that of carbohydrates^96,97^.

## Conclusion

Our findings demonstrate that mefenoxam induces significant taxonomic and functional shifts in the soil fungal communities even after a single application; however, the soil recovered within 12 weeks. Mefenoxam is widely used on different crops in the United States, with its recommended application frequency being every 14 days. Therefore, its effects on nontarget fungal communities are likely to be more pronounced in soils subject to frequent applications. Given this impact, the conventional agricultural practice of recurrent mefenoxam application is likely to exert considerable negative effects on the soil ecosystem, consequently affecting agricultural sustainability. There has to be judicious use of mefenoxam to minimize its harmful effects on nontarget fungal population and the emergence of resistant pathogen strains. Thus, we recommend further investigation in fields where mefenoxam is frequently used. This research greatly enhances our understanding of how mefenoxam application alters fungal community dynamics, their metabolic functions, and the subsequent implications for soil health and sustainable agriculture. This study aligned with the USDA policy on pesticide monitoring, which is aimed at ensuring sustainable agricultural practices.

### Supplementary Information

Supplementary Information.

## Data Availability

Thel raw fungal Illumina MiSeq sequences for this study are accessible on the NCBI Sequence Read Archive (SRA) repository with the bioproject number PRJNA1009143. For carrot cultivation samples, the SRA accession numbers are between SRR25758765 and SRR25758788, for no carrot cultivation, they range from SRR25759956 and SRR25759979.

## Abbreviations

CSS: Carrot cavity spot
CKNP: Neither Pythium inoculation nor mefenoxam application
CKP: Pythium inoculation without mefenoxam application
RNP: Mefenoxam application without Pythium inoculation
RP: Both Pythium inoculation and mefenoxam application
FLO: Fungal-like organisms
AWCD: Average well color development
USDA-NASS: USDA National Agricultural Statistics Service
