# Supplementary material for: Evaluating the effects of mefenoxam on taxonomic and functional dynamics of nontarget fungal communities during carrot cultivation
Source: Sci Rep. 2024 Apr 29;14:9867. doi: 10.1038/s41598-024-59587-2 (PMC11058253; doi:10.1038/s41598-024-59587-2)
Supplement: Supplementary file 1 — Supplementary Information. [file 41598_2024_59587_MOESM1_ESM.docx]

**Evaluating the Effects of Mefenoxam on Taxonomic and Functional Dynamics of Nontarget Fungal Communities during Carrot Cultivation**

Setu Bazie Tagele and Emma W. Gachomo*

Department of Microbiology and Plant Pathology, University of California–Riverside, Riverside, CA 92507, U.S.A.

***Correspondence**: Emma W. Gachomo (EWG); Email: emma.gachomo@ucr.edu; Tel.: +1 951-827-2093

# Supplementary Material

## Supplementary Figure

**
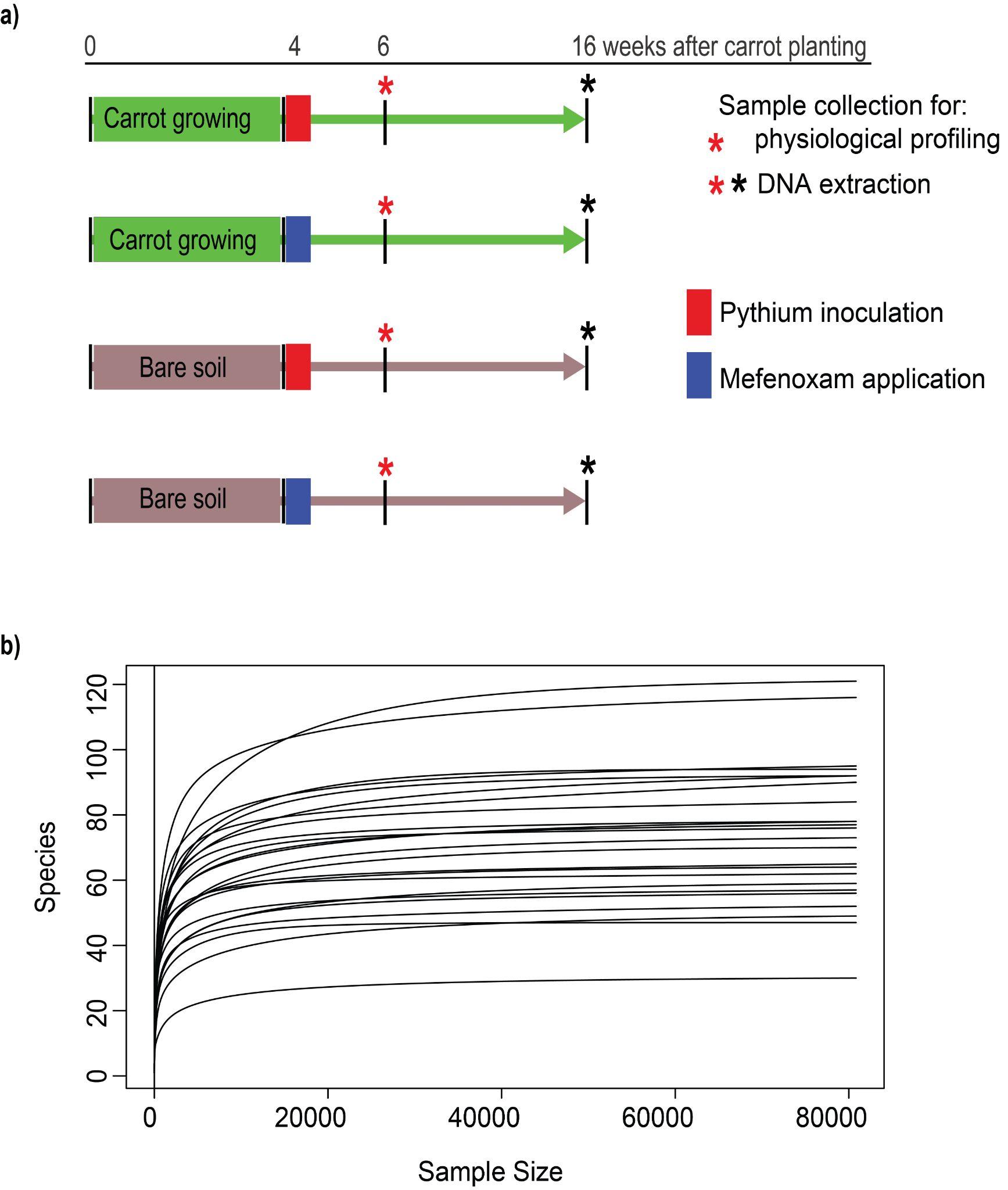
**

**Fig. S1:** A schematic illustration depicting the timing of treatment application and sample collection relative to carrot planting (a). Rarefaction curve depicting fungal observed species in 24 soil samples from the four treatments at two sampling times, T1 and T2 (b). T1 and T2 represent sampling times 2 and 12 weeks after treatment.


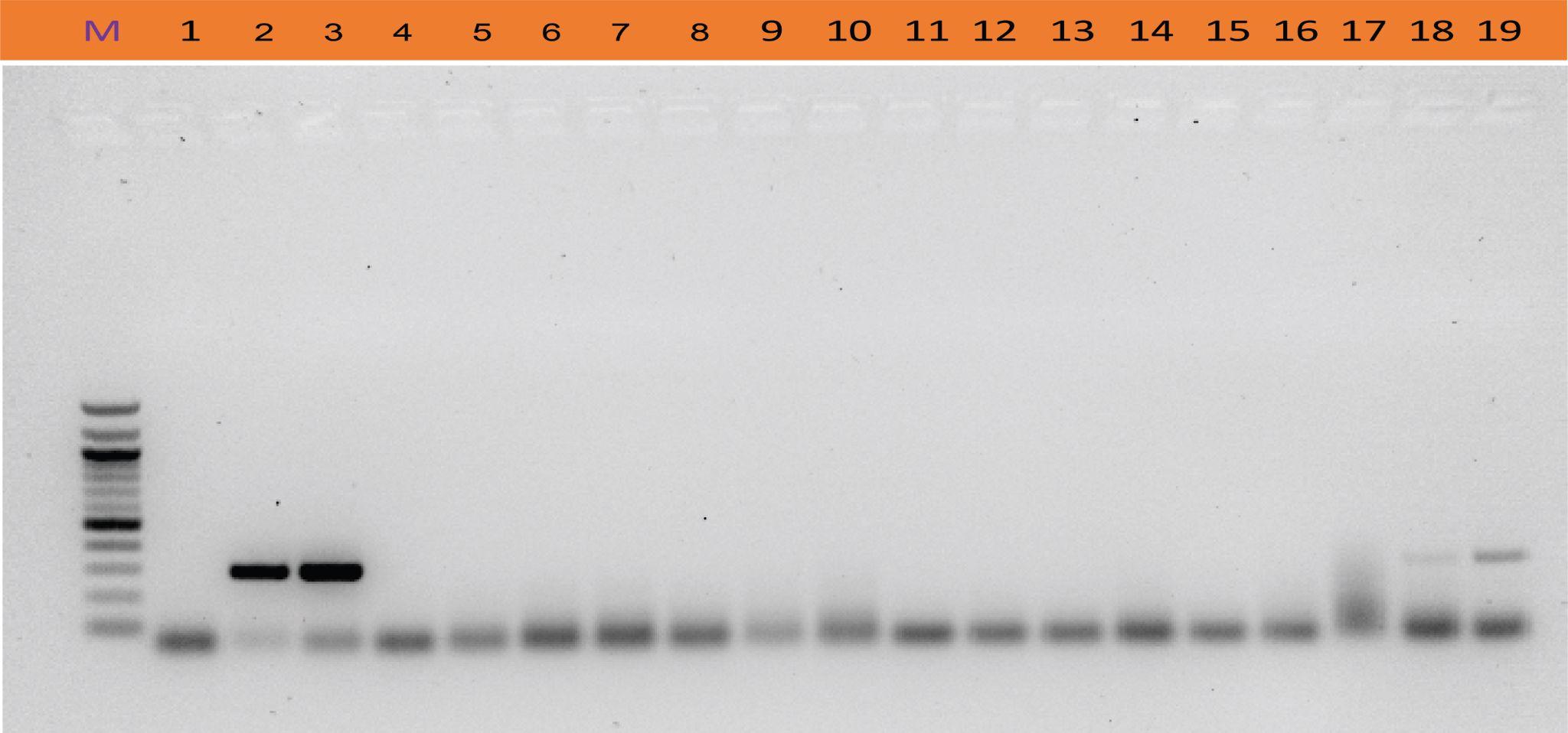


**Fig. S2: Agarose gel electrophresis of PCR product using *Mortierella alpina*-specific primers.** M: marker 100 bp DNA Ladder, Lane 1: negative control with no DNA, Lane 2: *M. alpina*  AD021, Lane 3: *M. alpina* AD072, Lane 4: *Mortierella polycephala* KOD948, Lane 5: *Mortierella* sp GBAus30, Lane 6: *Linnemannia elongata*, Lane 7: *Pythium irregulare*, CKNP from Lane 8 to Lane 10, CKP from Lane 11 to Lane 13, RNP from Lane 14 to Lane 16, and RP from Lane 17 to Lane 19. CKNP represents the control without *Pythium* inoculation or mefenoxam application, CKP represents *Pythium* inoculation without mefenoxam application, RNP represents mefenoxam application without *Pythium* inoculation, and RP represents *Pythium* inoculation and mefenoxam application. Original gel is presented in supplementary Fig. S2_Original_Gel.tif

**
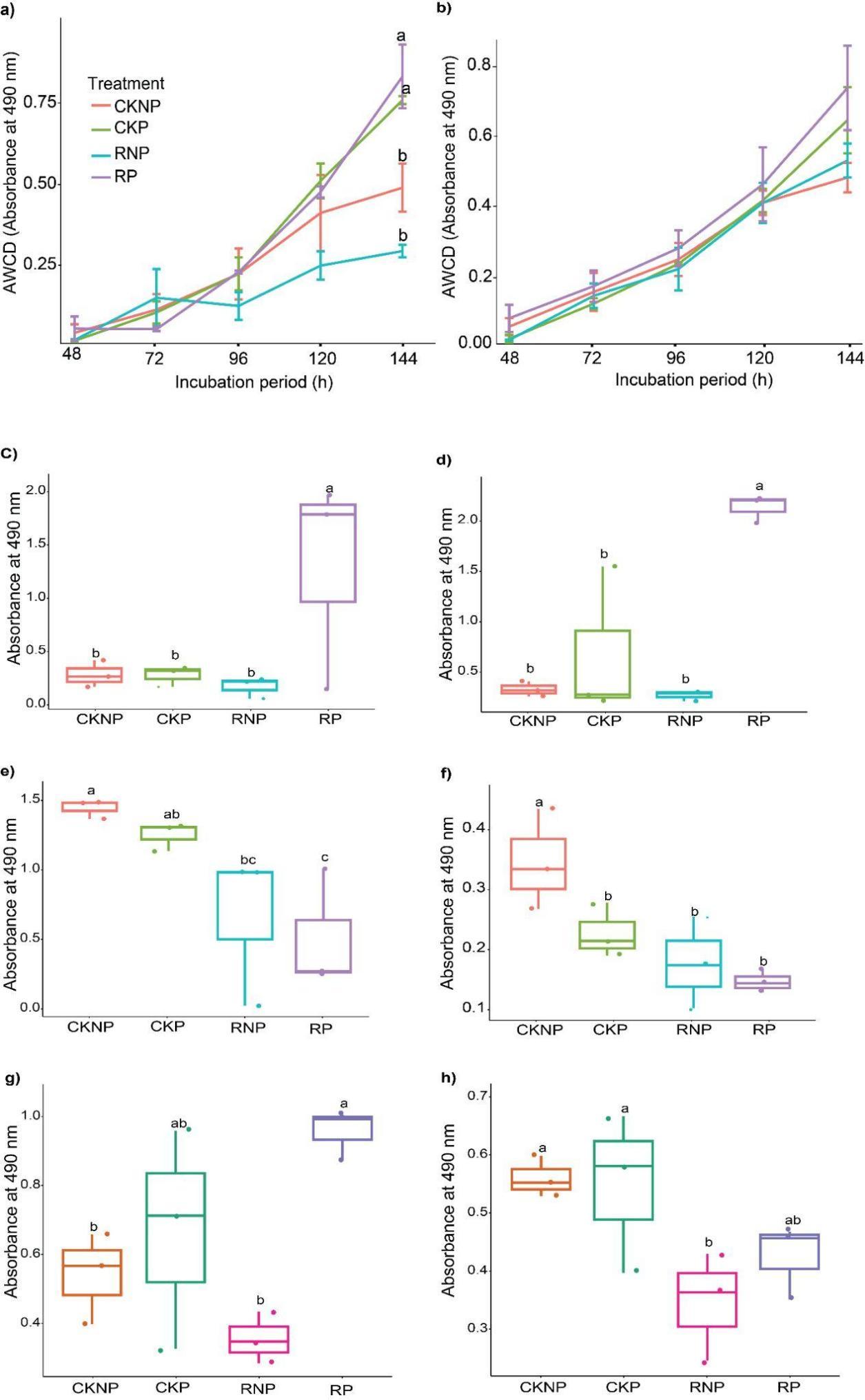
**

**Fig. S3: Carbon source utilization using Biolog FF plates.** a) Amines/amides and amino acids groups, b) polymer groups, c) arbutin, d) glycyl-L-glutamic acid, e) N-acetyl-D-galactosamine, f) glycyl-L-glutamic acid, g) Tween80, and h) β-cyclodextrin. CKNP represents the control without *Pythium* inoculation or mefenoxam application, CKP represents *Pythium* inoculation without mefenoxam application, RNP represents mefenoxam application without *Pythium* inoculation, and RP represents *Pythium* inoculation and mefenoxam application.

**
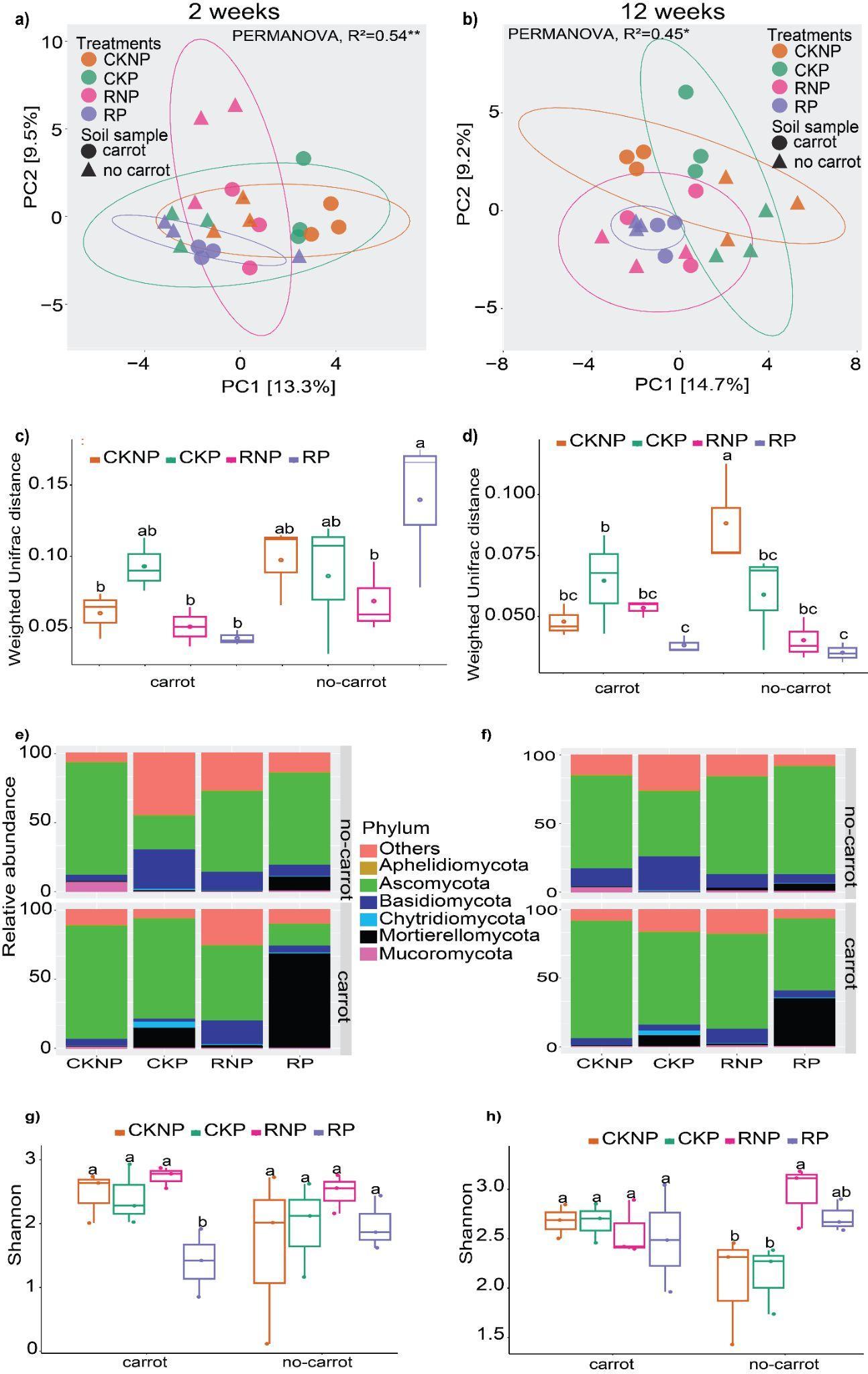
**

**Fig. S4: Fungal diversity and community structure.** Principal component analysis exhibiting beta diversity, measured based on UniFrac distances, at T1 (a) and T2 (b); ordination of treatments at T1 (c) and T2 (d); relative abundance of fungal phyla in different treatments at T1 (e) and T2 (f); and alpha diversity, Shannon, at T1 (g) and T2 (h). T1 and T2 represent sampling times 2 and 12 weeks after treatment, different letter(s) on the bar plot show significant differences among treatments at p < 0.05, CKNP represents the control without *Pythium* inoculation or mefenoxam application, CKP represents *Pythium* inoculation without mefenoxam application, RNP represents mefenoxam application without *Pythium* inoculation, and RP represents *Pythium* inoculation and mefenoxam application.

## Supplementary Tables

**Supplementary Table S1**: Chemicals sprayed in carrot fields in the United States of America between 2000 and 2020.

| Chemicals | 2000 | 2002 | 2004 | 2006 | 2010 | 2014 | 2016 | 2018 | 2020 |
| --- | --- | --- | --- | --- | --- | --- | --- | --- | --- |
| Azoxystrobin |  | √ |  | √ | √ | √ | √ | √ | √ |
| Boscalid |  |  |  |  | √ | √ | √ | √ | √ |
| Chlorothalonil | √ | √ | √ | √ | √ | √ | √ | √ | √ |
| Copper hydroxide | √ |  | √ | √ | √ |  |  | √ | √ |
| Cyazofamid |  |  |  |  |  |  | √ | √ | √ |
| Iprodione |  |  |  |  |  |  |  | √ |  |
| Mefenoxam | √ | √ | √ | √ | √ | √ | √ | √ | √ |
| Pyraclostrobin |  |  |  | √ | √ | √ | √ | √ | √ |
| Sulfur |  | √ |  | √ | √ | √ | √ | √ | √ |
| Penthiopyrad |  |  |  |  |  |  | √ | √ | √ |
| Propiconazole |  |  |  |  |  |  | √ | √ | √ |

**Source:** USDA agricultural stats [(“USDA - National Agricultural Statistics Service - Surveys - Agricultural Chemical Use Program” n.d.)](https://paperpile.com/c/EPQudR/P9K3L).

**Supplementary Table S2**: *Mortierella alpina*-specific primers and PCR condition

| Primer set | Sequences (5’-3’) | GC(%) | Tm(°C) | PCR reaction conditions | Amplicon size (bp) |
| --- | --- | --- | --- | --- | --- |
| MortF | CCCGATAACGAGAAGAGCGACA | 54.5 | 58.0 | Initial denaturation at 95℃ for 3; followed by 35 cycles at 95℃for 30 s, at 57℃for 30 s, at 72℃for 30 s; final elongation at 72℃ for 5 min. | 294 |
| MortR | CAGCGTCGCAGCGTTGATAATGT | 52.2 | 60.5 |  |  |

**Supplementary Table S3**: Two-way factorial ANOVA analysis to determine the effect of treatments and sampling time on fungal alpha diversity indices.

| Sampling time | Treatments | Alpha diversity indices | | | | | |
| --- | --- | --- | --- | --- | --- | --- | --- |
|  |  | Observed | Shannon | Simpson | InvSimpson | Fisher | PD |
| 2 weeks |  |  |  |  |  |  |  |
|  | CKNP | 65.3 ± 7.32 | 2.5 ± 0.23^a^ | 0.79 ± 0.07^a^ | 5.5 ± 1.24 | 7.0 ± 0.62 | 20.8 ± 1.58 |
|  | CKP | 70.7 ± 13.88 | 2.5 ± 0.28^a^ | 0.83 ± 0.05^a^ | 7 ± 2.2 | 7.7 ± 1.42 | 22.5 ± 2.79 |
|  | RNP | 78.3 ± 9.5 | 2.8 ± 0.09^a^ | 0.89 ± 0.02^a^ | 9.6 ± 0.94 | 8.6 ± 1.05 | 25.4 ± 1.96 |
|  | RP | 45.0 ± 8.33 | 1.4 ± 0.31^b^ | 0.52 ± 0.13^b^ | 2.4 ± 0.56 | 4.6 ± 0.87 | 19.4 ± 2.52 |
|  | Significance level | ns | * | * | ns | ns | ns |
| 12 weeks |  |  |  |  |  |  |  |
|  | CKNP | 100.3 ± 8.51 | 2.9 ± 0.07 | 0.87 ± 0.01 | 7.7 ± 0.61 | 11.3 ± 0.82 | 26.5 ± 0.48 |
|  | CKP | 76.0 ± 8.84 | 2.6 ± 0.1 | 0.87 ± 0.02 | 8 ± 0.76 | 8.3 ± 1.08 | 23.6 ± 1.42 |
|  | RNP | 83.7 ± 19.86 | 2.6 ± 0.17 | 0.87 ± 0.03 | 8.4 ± 1.8 | 9.3 ± 2.17 | 22.5 ± 2.5 |
|  | RP | 73.0 ± 3.67 | 2.6 ± 0.32 | 0.82 ± 0.08 | 8.1 ± 3.08 | 7.9 ± 0.51 | 20.3 ± 1.06 |
|  | Significance level | ns | ns | ns | ns | ns | ns |

Note: Separate mean comparison was performed for each sampling time. Different letter (s) denote statistically significant differences (p < 0.05) between treatments. *significant (p < 0.05) and ns – non significant. CKNP represents the control without *Pythium* inoculation or mefenoxam application, CKP represents *Pythium* inoculation without mefenoxam application, RNP represents mefenoxam application without *Pythium* inoculation, and RP represents *Pythium* inoculation and mefenoxam application.

**Supplementary Table S4:** Two-way factorial ANOVA analysis to determine the impacts of treatments and sampling time on fungal communities at the phylum level.

| Sampling time | Treatments | Relative abundance (%) | | | | | |
| --- | --- | --- | --- | --- | --- | --- | --- |
|  |  | Ascomycota | Mortierellomycota | Basidiomycota | Chytridiomycota | Mucoromycota | Others |
| 2 weeks |  |  |  |  |  |  |  |
|  | CKNP | 82.3 ± 6.81^a^ | 0.1 ± 0.01^b^ | 5 ± 1.88^b^ | 0.1 ± 0.05 | 1.5 ± 0.69 | 11.3 ± 5.73^b^ |
|  | CKP | 72.7 ± 14.19^a^ | 14.5 ± 14.37^b^ | 2.2 ± 0.66^b^ | 4.5 ± 4.48 | 0.2 ± 0.16 | 6.1 ± 1.45^b^ |
|  | RNP | 54.5 ± 6.01^a^ | 1.8 ± 1.78^b^ | 17.3 ± 5.54^a^ | 0.6 ± 0.53 | 0.3 ± 0.08 | 25.7 ± 1.97^a^ |
|  | RP | 16.1 ± 6.03^b^ | 68.5 ± 9.78^a^ | 5 ± 2.36^b^ | 0.2 ± 0.14 | 0.2 ± 0.11 | 10.3 ± 4.22^b^ |
|  | Significance level | ** | ** | * | ns | ns | ns |
| 12 weeks |  |  |  |  |  |  |  |
|  | CKNP | 91.8 ± 1.03 | 0.1 ± 0.01^b^ | 4.1 ± 1.26 | 0.6 ± 0.52 | 0.5 ± 0.11 | 3.2 ± 0.45 |
|  | CKP | 71.9 ± 11.09 | 0.1 ± 0.03^b^ | 19.1 ± 11.52 | 0.1 ± 0.01 | 0.2 ± 0.12 | 9 ± 4.88 |
|  | RNP | 84.5 ± 4.76 | 0.3 ± 0.29^b^ | 4 ± 1.86 | 0.3 ± 0.13 | 1.4 ± 1.16 | 9.6 ± 2.19 |
|  | RP | 89.9 ± 1.98 | 2.2 ± 0.48^a^ | 4.9 ± 2.65 | 0.2 ± 0.1 | 0.8 ± 0.17 | 2.3 ± 0.72 |
|  | Significance level | ns | ns | ns | ns | ns | ns |

Note: Separate mean comparison was performed for each sampling time. Different letter (s) denote statistically significant differences (p < 0.05) between treatments. **significant (p < 0.01), *significant (p < 0.05) and ns – non significant. CKNP represents the control without *Pythium* inoculation or mefenoxam application, CKP represents *Pythium* inoculation without mefenoxam application, RNP represents mefenoxam application without *Pythium* inoculation, and RP represents *Pythium* inoculation and mefenoxam application.

**Supplementary Table S5:** Two-way factorial ANOVA to determine the impacts of treatments and sampling time on fungal communities at the class level

| Class | Relative abundance (%) | | | | | | | | | | |
| --- | --- | --- | --- | --- | --- | --- | --- | --- | --- | --- | --- |
|  | 2 weeks | | | | |  | 12 weeks | | | | |
|  | CKNP | CKP | RNP | RP | Significance level |  | CKNP | CKP | RNP | RP | Significance level |
| Eurotiomycetes | 16.7 ± 5^a^ | 23.4 ± 13^a^ | 11.8 ± 1^a^ | 1 ± 0.4^b^ | ** |  | 13.6 ± 3^bc^ | 2.7 ± 0.7^c^ | 21.1 ± 3^ab^ | 25.5 ± 7^a^ | * |
| Mortierellomycetes | 0.1 ± 0.01^b^ | 14.5 ± 14^b^ | 1.8 ± 1.7^b^ | 68.4 ± 10^a^ | ** |  | 0.1 ± 0.01 | 0.1 ± 0.03 | 0.3 ± 0.3 | 2.2 ± 0.5 | ns |
| Sordariomycetes | 13.3 ± 2^a^ | 12.1 ± 4^a^ | 4 ± 0.5^b^ | 1.1 ± 0.5^b^ | * |  | 52.5 ± 6 | 45.4 ± 11 | 46 ± 7 | 22.8 ± 6 | ns |
| Agaricomycetes | 2.4 ± 0.2^b^ | 1.9 ± 0.5^b^ | 17.3 ± 5.5^a^ | 5.1 ± 2.4^b^ | * |  | 2.8 ± 0.6 | 5.1 ± 2.9 | 2.1 ± 0.9 | 4.8 ± 2.6 | ns |
| Leotiomycetes | 24.6 ± 19 | 16.4 ± 8 | 5.2 ± 0.8 | 2 ± 1 | ns |  | 8.6 ± 4 | 1.9 ± 0.7 | 7.6 ± 4 | 30.7 ± 14 | ns |
| Pezizomycetes | 24.8 ± 2 | 8.9 ± 2 | 7 ± 0.6 | 5.5 ± 4 | ns |  | 8.6 ± 3 | 16.1 ± 4 | 9.2 ± 7 | 8.3 ± 6 | ns |
| Dothideomycetes | 3 ± 1.0 | 12.1 ± 11 | 25.5 ± 5 | 6.8 ± 2 | ns |  | 8.5 ± 6 | 6 ± 5 | 0.7 ± 0.3 | 2.4 ± 1.8 | ns |
| Ustilaginomycetes | 2.2 ± 2.1 | 0.1 ± 0.03 | 0.1 ± 0.01 | 0.1 ± 0.01 | ns |  | 0.7 ± 0.38 | 13.7 ± 13.4 | 2 ± 1.88 | 0.1 ± 0.09 | ns |
| Spizellomycetes | 0 ± 0 | 4.5 ± 4.48 | 0.5 ± 0.44 | 0.2 ± 0.16 | ns |  | 0.5 ± 0.47 | 0.1 ± 0.01 | 0.2 ± 0.12 | 0 ± 0 | ns |
| Umbelopsidomycetes | 1 ± 0.81 | 0.2 ± 0.17 | 0.2 ± 0.1 | 0.1 ± 0.08 | ns |  | 0.4 ± 0.1 | 0.2 ± 0.14 | 1.4 ± 1.19 | 0.7 ± 0.21 | ns |
| Microbotryomycetes | 0.2 ± 0.12 | 0.1 ± 0.1 | 0.1 ± 0.02 | 0 ± 0 | ns |  | 0.5 ± 0.48 | 0.1 ± 0.1 | 0 ± 0 | 0.1 ± 0.01 | ns |
| Mucoromycetes | 0.6 ± 0.57 | 0.1 ± 0.01 | 0.1 ± 0.04 | 0.1 ± 0.04 | ns |  | 0.1 ± 0.04 | 0 ± 0 | 0.1 ± 0.03 | 0.1 ± 0.05 | ns |
| Chytridiomycetes | 0.1 ± 0.06 | 0.1 ± 0.01 | 0.2 ± 0.11 | 0.1 ± 0.05 | ns |  | 0.2 ± 0.06 | 0.1 ± 0.02 | 0.2 ± 0.13 | 0.2 ± 0.1 | ns |
| Saccharomycetes | 0 ± 0 | 0.1 ± 0.03 | 0 ± 0 | 0 ± 0 | ns |  | 0.2 ± 0.15 | 0.1 ± 0.02 | 0.1 ± 0.03 | 0.5 ± 0.42 | ns |
| Others | 11.8 ± 5.77^b^ | 6.3 ± 1.4^b^ | 27 ± 2.69^a^ | 10.4 ± 4.18^b^ | * |  | 3.4 ± 0.41^b^ | 9.2 ± 4.73^b^ | 9.9 ± 2.4^b^ | 2.3 ± 0.73^b^ | * |

Note: Separate mean comparison was performed for each sampling time. Different letter (s) denote statistically significant differences (p < 0.05) between treatments. **significant (p < 0.01), *significant (p < 0.05) and ns – non significant. CKNP represents the control without *Pythium* inoculation or mefenoxam application, CKP represents *Pythium* inoculation without mefenoxam application, RNP represents mefenoxam application without *Pythium* inoculation, and RP represents *Pythium* inoculation and mefenoxam application.

**Supplementary Table S6:** Two-way factorial ANOVA to determine the impacts of treatments and sampling time on fungal communities at the genus level

| Class | Relative abundance (%) | | | | | | | | | | |
| --- | --- | --- | --- | --- | --- | --- | --- | --- | --- | --- | --- |
|  | 2 weeks | | | | |  | 12 weeks | | | | |
|  | CKNP | CKP | RNP | RP | Significance level |  | CKNP | CKP | RNP | RP | Significance level |
| *Penicillium* | 16.3 ± 5.4^a^ | 22.2 ± 12.7^a^ | 11.6 ± 1.2^a^ | 0.8 ± 0.3^b^ | ** |  | 9.8 ± 2.3^bc^ | 2.3 ± 0.7^c^ | 20.7 ± 3.4^ab^ | 25.0 ± 7.6^a^ | * |
| *Mortierella* | 0.1 ± 0.01^b^ | 14.5 ± 14^b^ | 1.9 ± 1.8^b^ | 68.6 ± 9.7^a^ | ** |  | 0 ± 0 | 1.9 ± 1.6 | 0.3 ± 0.3 | 1.7 ± 0.9 | ns |
| *Trichoderma* | 5.5 ± 2.1^a^ | 6.2 ± 1.4^a^ | 1.1 ± 0.3^b^ | 0.1 ± 0.06^b^ | * |  | 28.4 ± 13.0 | 9.2 ± 0.8 | 7.8 ± 6.3 | 8 ± 3.8 | ns |
| *Alternaria* | 1.7 ± 0.9^b^ | 6.9 ± 6.8^b^ | 24.5 ± 4.6^a^ | 6.2 ± 2.1^b^ | * |  | 5.2 ± 3.4 | 6.6 ± 3.2 | 0.3 ± 0.1 | 1.7 ± 1.6 | ns |
| *Acremonium* | 0.5 ± 0.5 | 0.2 ± 0.15 | 0.1 ± 0.01 | 0.1 ± 0.01 | ns |  | 13 ± 10.3 | 4.2 ± 2.4 | 7.8 ± 3.5 | 3.7 ± 2.5 | ns |
| *Chromelosporium* | 24.9 ± 10.9 | 8.9 ± 1.8 | 6.7 ± 0.7 | 5.3 ± 3.6 | ns |  | 7 ± 1.9 | 14.4 ± 4.1 | 9.3 ± 7.2 | 8.3 ± 6.3 | ns |
| *Sarocladium* | 0.1 ± 0.09 | 0.2 ± 0.16 | 0.2 ± 0.05 | 0 ± 0 | ns |  | 1.1 ± 0.3 | 4.5 ± 2.3 | 5.3 ± 2.5 | 3.8 ± 2.2 | ns |
| *Plectosphaerella* | 0.1 ± 0.08 | 0.1 ± 0.01 | 0.4 ± 0.34 | 0 ± 0 | ns |  | 0.8 ± 0.7 | 4.7 ± 4.7 | 12.2 ± 11.8 | 0.5 ± 0.3 | ns |
| *Fusarium* | 3.5 ± 0.9^a^ | 0.4 ± 0.3^b^ | 0.3 ± 0.1^b^ | 0 ± 0^b^ | ** |  | 6.9 ± 2.7 | 1.2 ± 1.0 | 0.3 ± 0.1 | 3.3 ± 3.3 | ns |
| *Cladosporium* | 1.4 ± 0.4 | 3.7 ± 3.6 | 0.6 ± 0.3 | 0.5 ± 0.4 | ns |  | 4.6 ± 2.8 | 3.2 ± 2.7 | 0.4 ± 0.2 | 0.8 ± 0.4 | ns |

Note: Separate mean comparison was performed for each sampling time. Different letter (s) denote statistically significant differences (p < 0.05) between treatments. **significant (p < 0.01), *significant (p < 0.05) and ns – non significant. CKNP represents the control without *Pythium* inoculation or mefenoxam application, CKP represents *Pythium* inoculation without mefenoxam application, RNP represents mefenoxam application without *Pythium* inoculation, and RP represents *Pythium* inoculation and mefenoxam application.
